# Complement factor H in cancer biology: intracellular functions, tumor–host interactions, and systemic context

**DOI:** 10.3389/fimmu.2026.1877625

**Published:** 2026-07-29

**Authors:** Xitan Wang, Meiying Mu, Han Li

**Affiliations:** Department of Oncology, Zibo Central Hospital, Zibo, China

**Keywords:** anti-FH autoantibodies, biomarkers, CAPZ, complement Factor H, E2F3, GT103, intracellular FH, systemic context

## Abstract

Complement factor H (FH), encoded by CFH, is a principal soluble regulator of the alternative complement pathway, but its roles in cancer extend beyond extracellular complement control. Intracellular FH has been linked to p53/NF-κB signaling, cell-cycle regulation, and cytoskeletal organization. Recent studies distinguish a nuclear FH–E2F3 interaction associated with p53 and cell-cycle control from a cytosolic FH–CapZ-associated mechanism involved in actin organization. Although FH has been detected in lysosome-positive compartments, current evidence does not establish lysosomes as the functional site of these intracellular effects. At the tumor–host interface, extracellular FH protects tumor-cell and extracellular-vesicle surfaces from complement-mediated injury and phagocytic clearance. Locally produced FH and FHL-1 have also been associated with distinct myeloid-cell and regulatory T-cell programs, including direct FH–ICOS engagement. Structurally altered tumor-associated FH can become a target of endogenous anti-FH antibodies and of the therapeutic antibody GT103, linking local complement regulation with humoral recognition and therapeutic intervention. Across tissues and circulation, FH exists in intracellular, soluble, surface-associated, and extracellular-vesicle-associated pools shaped by hepatic production, local synthesis, surface recruitment, distribution, and clearance. These pools are not interchangeable: their functions and clinical associations depend on both source and compartment. We therefore propose a source- and compartment-resolved framework that separates systemic FH availability from local intracellular and extracellular functions, while treating cross-compartment relationships as testable rather than established causal sequences. Tissue and circulating FH measurements are being explored as context-dependent biomarkers, and GT103 has progressed through phase Ib and completed phase II evaluation in refractory non-small cell lung cancer. Further progress will require tissue-specific experimental models, paired tissue and circulating measurements, clearer mapping of intracellular interaction sites, and biomarker analyses that account separately for hepatic production, inflammatory state, renal handling or protein loss, and altered distribution or clearance.

## 1 Introduction

The complement system is a distributed network comprising soluble components, membrane-associated receptors and regulators, and intracellular complement-related molecules (1–4). Its extracellular pathways recognize altered or foreign surfaces, promote opsonization and inflammatory signaling, and may culminate in membrane attack complex formation (1, 2). In cancer, however, complement activity is highly context dependent. Complement-mediated cytotoxicity and immune surveillance may contribute to tumor control, whereas persistent or spatially restricted activation can promote inflammation, angiogenesis, stromal remodeling, and immunosuppression (5–8). The intracellular complement system, commonly referred to as the *complosome*, regulates cellular metabolism and immune-cell function, extending complement biology beyond the traditional extracellular cascade (3, 9). Complement activity may also be altered by anticancer treatment. For example, neoadjuvant therapy has been associated with changes in complement signaling within the pancreatic cancer microenvironment, illustrating the need to interpret complement-related findings in relation to tissue context, disease stage, and therapeutic exposure (10).

Complement factor H (FH), encoded by CFH, is a 155-kDa glycoprotein composed of 20 complement control protein domains, also termed short consensus repeats (SCRs) (11, 12). FH is a principal soluble regulator of the alternative complement pathway. Its canonical activities include accelerating the decay of the existing C3 convertase C3bBb, acting as a cofactor for factor I-mediated cleavage of C3b, and competing with factor B to limit the assembly of new convertases (11, 12). Binding to host-cell sialic acids and glycosaminoglycans, particularly through its C-terminal SCR domains, localizes these regulatory activities to self surfaces (11, 12). Factor H-like protein 1 (FHL-1) is an alternatively spliced product of CFH comprising the first seven SCR domains and a unique C-terminal sequence. By contrast, CFHR1–5 are separate homologous proteins that can compete with or otherwise modify FH-dependent complement regulation (12–14). Several CFHR proteins can reduce FH-mediated regulation by competing for shared ligands, thereby increasing C3b deposition, although individual CFHR proteins may also exert additional complement-modulating activities (13, 14). These canonical extracellular activities provide the biochemical basis of FH function but do not encompass the broader range of cell-intrinsic and tumor-associated activities now reported in cancer.

CFH dysfunction is well established in non-malignant disorders, including age-related macular degeneration, atypical hemolytic uremic syndrome, and C3 glomerulopathy (15–18). These diseases illustrate that FH activity depends not only on protein abundance but also on cellular source, local concentration, molecular interactions, surface recognition, and tissue environment. The same considerations apply in cancer. FH may be synthesized locally by tumor cells, stromal cells, or other tissue-resident cells; recruited from plasma to tumor-cell surfaces or extracellular vesicles; or detected within intracellular compartments (19–23). Distinguishing among these sources and compartments is important because extracellularly recruited FH, locally secreted FH, and intracellular FH may exert biologically distinct effects.

Cancer-associated FH biology therefore extends beyond extracellular complement evasion. Intracellular FH has been associated with the regulation of cell-cycle progression, cell survival, and actin-cytoskeleton organization (19, 20). Locally produced or recruited FH can modify complement activity within the tumor microenvironment and has been linked to selected myeloid-cell and regulatory T-cell programs (24, 25). In parallel, circulating FH, anti-FH autoantibodies, and FH-related proteins have been investigated as diagnostic or prognostic biomarkers in several cancers (26–30). The tumor-reactive anti-FH antibody GT103 has undergone phase Ib monotherapy evaluation, and results from a phase II study of GT103 combined with pembrolizumab in refractory metastatic non-small cell lung cancer have now been reported, providing initial clinical evidence that tumor-associated FH can be therapeutically targeted (31, 32).

**Figure 1:**
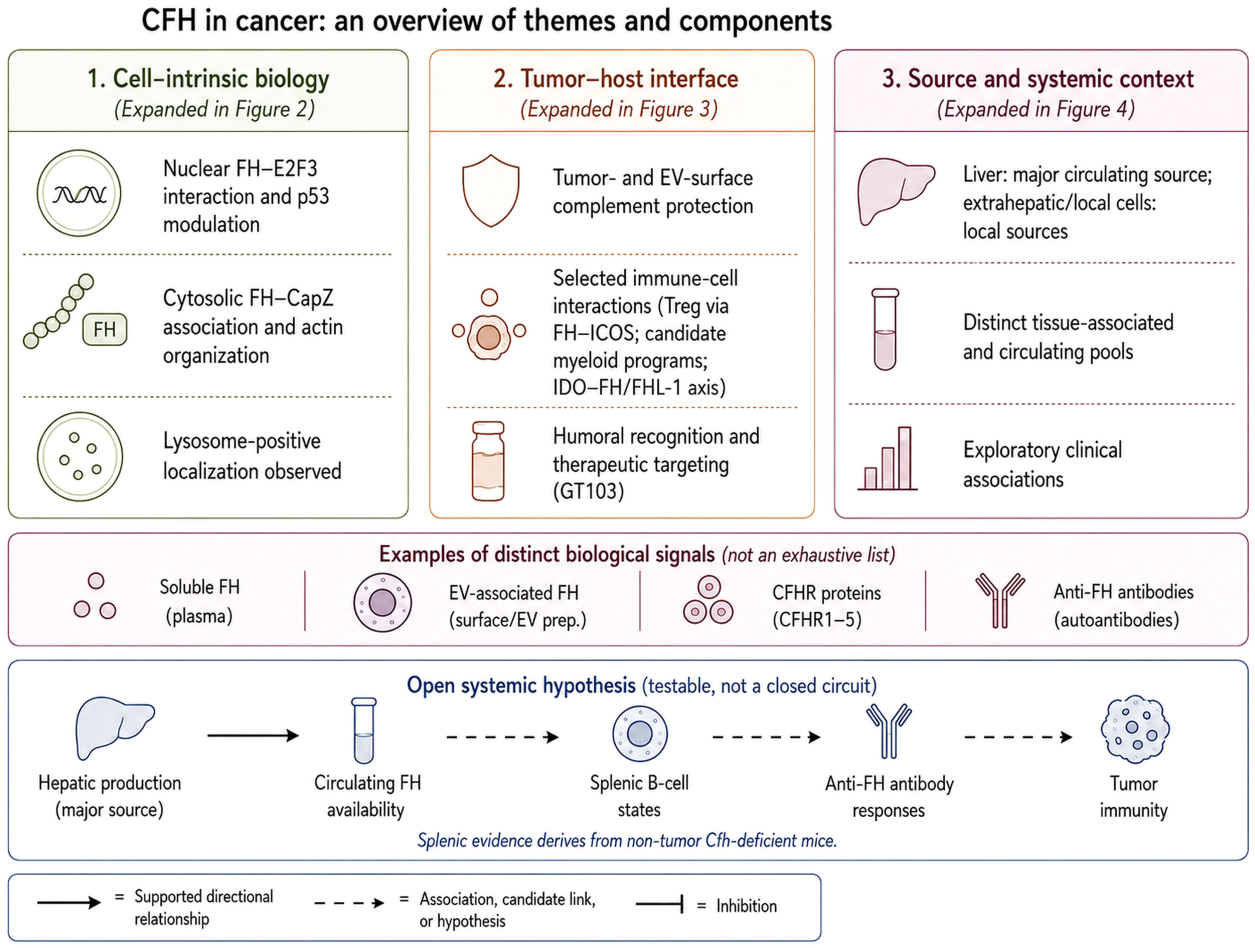
Overview of FH biology in cancer. The review is organized into three related but mechanistically distinct domains: cell-intrinsic biology, the tumor–host interface, and source/systemic context. The first domain distinguishes nuclear FH–E2F3 interactions, cytosolic FH–CAPZB/CapZ-associated biology, and lysosome-positive localization. The second covers tumor- and extracellular-vesicle (EV)-surface complement protection, selected immune-cell interactions, humoral recognition, and GT103 targeting. The third distinguishes hepatic and extrahepatic sources, tissue-associated and circulating pools, and exploratory clinical associations. Soluble FH, EV-associated FH, CFHR proteins, and anti-FH antibodies are shown as biologically distinct signals. The liver-to-circulating-FH relationship is supported, whereas the proposed links to splenic B-cell states, anti-FH responses, and tumor immunity constitute an open, testable hypothesis. Splenic evidence derives from non-tumor Cfh-deficient mice. Solid arrows indicate supported directional relationships; dashed arrows indicate associations, candidate links, or hypotheses; T-shaped lines indicate inhibition.

This review examines FH in cancer according to its cellular source, biological compartment, and mode of action. It first considers intracellular FH functions, including nuclear FH–E2F3 signaling and cytosolic FH–CapZ-associated regulation, and then examines extracellular FH at the tumor–host interface, including local complement protection, immune-cell interactions, humoral recognition, and therapeutic targeting. Tissue expression, circulating and extracellular-vesicle-associated FH, CFHR proteins, and anti-FH antibodies are subsequently discussed in relation to their biological context and biomarker potential. Evidence concerning hepatic production, circulating FH availability, splenic B-cell biology, and tumor-associated FH is finally integrated into a source- and compartment-resolved systemic hypothesis. Because these observations derive from different tumor types, non-tumor models, and experimental settings, the proposed connections are treated as testable relationships rather than as a continuous causal pathway. **Figure 1** provides an overview of these three analytical domains and distinguishes supported relationships from hypothesis-generating links. Terminology and operational distinctions among FH, FHL-1, CFHR1–5, EV-associated FH, anti-FH antibodies, and GT103 are summarized in **Table 1**.

**Table 1:** Operational definitions for FH-related entities and compartments discussed in this review.

Term/entity	Definition and source	Functional or clinical meaning	Key interpretive caution and ref(s)
Canonical extracellular FH	Full-length soluble FH protein, encoded by CFH and produced mainly by the liver but also by extrahepatic cells.	Regulates the alternative pathway through decay acceleration of C3bBb, factor I cofactor activity, and limitation of new convertase assembly on self surfaces.	Protein abundance alone does not specify its cellular source or surface context (11–14).
Intracellular FH	FH detected within tumor or non-tumor cells, including nuclear and cytosolic pools.	Associated with p53/E2F3-dependent cell-cycle control and CapZ-associated actin organization.	Lysosome-positive localization does not define a lysosomal mechanism; intracellular trafficking and exact interface residues remain unresolved (19, 20).
Locally synthesized FH	FH produced by tumor, stromal, endothelial, epithelial, or immune cells within a tissue.	May contribute to local complement regulation or cell-intrinsic and immune-cell effects.	Tissue protein detection cannot distinguish local synthesis from deposition unless paired with transcriptional or source-resolved evidence (14, 19, 23–25, 33, 34, 47).
Surface-recruited FH	Circulating or locally secreted FH bound to tumor-cell or other extracellular surfaces.	Protects surfaces from alternative-pathway amplification and may expose tumor-associated epitopes.	Surface abundance is not equivalent to CFH transcription in the same cell (21, 46).
EV-associated FH	FH detected on or within extracellular vesicle preparations.	May protect tumor-derived EVs from complement-mediated lysis and phagocytosis and can serve as a compartment-specific biomarker.	Requires methods that distinguish genuine vesicular association from residual soluble plasma FH (21, 28).
FHL-1	Alternative splice product of CFH containing CCP1–7 plus a unique C-terminal sequence.	Retains N-terminal complement-regulatory functions and has been linked to tumor-associated immune effects in selected models.	FHL-1 is not a truncated degradation product and should not be treated as interchangeable with full-length FH (12–14, 24, 33).
CFHR1–5	Five separate homologous proteins encoded by CFHR genes.	Can compete with or otherwise modify FH-dependent regulation and show tumor-specific expression associations.	Family members are biochemically distinct; tissue transcript changes do not directly indicate circulating protein function (13, 14, 54–60).
Endogenous anti-FH autoantibodies	Host antibodies recognizing native or structurally altered FH epitopes.	Associated with early-stage NSCLC or favorable outcome in selected cohorts and may reflect humoral recognition of tumor-associated FH.	Antibody positivity does not measure circulating FH abundance; epitope, assay, and disease context differ across studies (26, 27, 49, 50).
GT103	Fully human IgG3 monoclonal antibody derived from patient B cells and directed against a tumor-associated conformational FH epitope.	Activates complement/Fc-dependent antitumor mechanisms and has completed phase Ib and phase II evaluation in refractory NSCLC.	Targets an accessible extracellular FH compartment and is not expected to directly modify nuclear or cytosolic FH functions (21, 31, 32, 46, 51).

CCP, complement control protein domain; EV, extracellular vesicle; FH, factor H; FHL-1, factor H-like protein 1; CFHR, factor H-related protein; NSCLC, non-small cell lung cancer. This table is an operational glossary; the categories describe source and compartment and are not intended as equivalent mechanistic tiers.

## 2 Cell-intrinsic functions and regulation of FH

**Figure 2:**
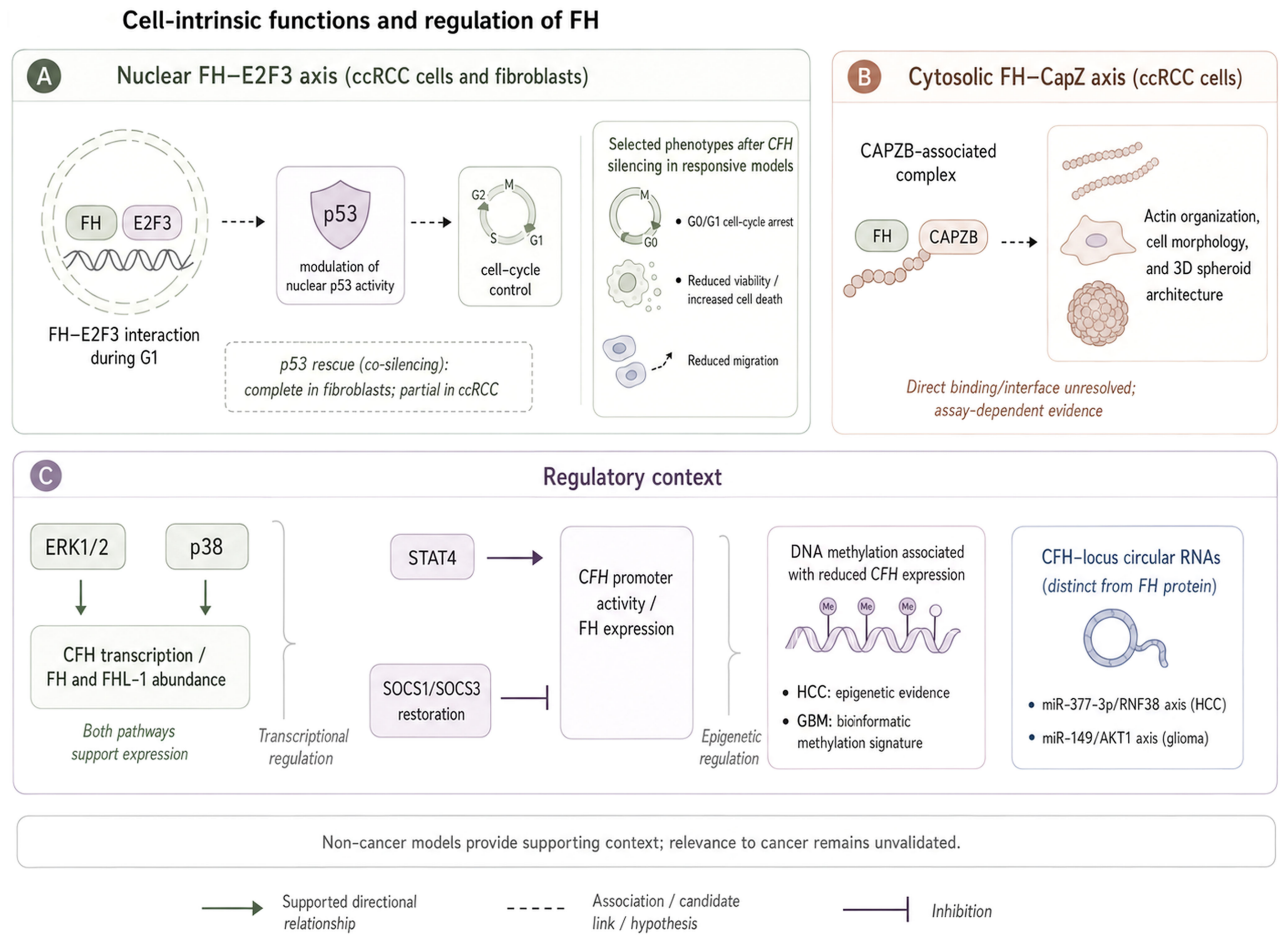
Cell-intrinsic functions and regulation of FH. **(A)** Nuclear FH-E2F3 axis. In ccRCC cells and fibroblasts, FH interacts with E2F3 during G1. Downstream modulation of nuclear p53 activity and cell-cycle control is presented as a working model. In selected responsive models, CFH silencing is associated with G0/G1 arrest, reduced viability or increased cell death, and reduced migration. Co-silencing of p53 completely reverses these phenotypes in fibroblasts and partially reverses them in ccRCC cells. **(B)** Cytosolic FH-CapZ axis. In ccRCC cells, cytosolic FH is associated with CAPZB/CapZ and with actin organization, cell morphology, and three-dimensional spheroid architecture; the direct binding interface remains unresolved and the evidence is assay dependent. **(C)** Regulatory context. ERK1/2 and p38 support CFH transcription and FH/FHL-1 abundance. STAT4 activates CFH promoter activity, whereas SOCS1/SOCS3 suppress this output. Additional regulatory contexts include CFH methylation-associated evidence in HCC and GBM and CFH-locus circular RNAs acting through the miR-377-3p/RNF38 axis in HCC and the miR-149/AKT1 axis in glioma. Non-cancer models provide supporting biological context, but their relevance to cancer remains unvalidated. Solid arrows indicate supported directional relationships; dashed arrows indicate associations, candidate links, or hypotheses; T-shaped lines indicate inhibition.

The principal cell-intrinsic phenotypes, spatially distinct nuclear and cytosolic mechanisms, and regulatory contexts discussed in this section are summarized in **Figure 2**.

### 2.1 Intracellular FH, p53/NF-κB, and localization

Daugan and colleagues identified intracellular FH in clear-cell renal cell carcinoma (ccRCC) and lung cancer models (19). In ccRCC cell lines, CFH silencing reduced proliferation and migration, increased cell death, and induced cell-cycle arrest, accompanied by increased p53 phosphorylation and NF-κB nuclear translocation (19). An FH-dependent phenotype was also observed in a lung adenocarcinoma model, whereas the tested lung squamous carcinoma cell line, human umbilical vein endothelial cells, and proximal tubular epithelial cells showed no comparable response to CFH silencing (19).

In tissue sections, FH was detected in lysosome-positive intracellular compartments and co-distributed with C3 (19). The functional experiments, however, did not localize the p53/NF-κB phenotype specifically to lysosomes or link it to FH-dependent regulation of intracellular C3 activation. The findings are therefore most accurately interpreted as evidence for a cell-type-dependent intracellular FH function.

### 2.2 Nuclear FH–E2F3 interactions and cell-cycle regulation

Rezola Artero and colleagues detected nuclear FH in ccRCC cells and fibroblasts and demonstrated its interaction with the cell-cycle transcription factor E2F3 (20). CFH silencing increased nuclear p53 accumulation, activation, and transcriptional activity, reduced cell proliferation and viability, and promoted G0/G1 arrest (20). Co-silencing of p53 reversed these effects completely in fibroblasts and partially in ccRCC cells, supporting a model in which FH–E2F3 interactions during G1 influence nuclear p53 activity.

Surface plasmon resonance analysis identified CCP6–8 as the principal E2F3-binding region of FH under physiological ionic-strength conditions, although the amino-acid residues forming the interface have not been mapped (20). A putative nuclear localization signal was computationally predicted in the linker between CCP1 and CCP2 (20). Intracellular FH also migrated at a lower apparent molecular mass than secreted or plasma-derived FH. The magnitude of this difference was similar to that observed after enzymatic deglycosylation of plasma FH, consistent with a predominantly non-glycosylated intracellular form (20).

### 2.3 Cytosolic FH, CAPZB, and actin organization

The cytoskeletal effects of FH were associated with the actin-capping complex rather than with its nuclear interaction with E2F3. Proteomic analysis and co-immunoprecipitation identified CAPZB and other components of the CapZ complex among FH-associated proteins in ccRCC cells (20). Silencing either CFH or CAPZB altered cell morphology and three-dimensional spheroid organization, whereas combined silencing produced no clear additive effect, consistent with their participation in a shared cytoskeletal process (20).

Interaction assays performed under low-ionic-strength conditions implicated CCP6–8 and the C-terminal CCP18–20/CCP19–20 regions in CAPZB binding (20). CAPZB binding was not detected by surface plasmon resonance under physiological ionic-strength conditions, although an ELISA-based assay showed a dose-dependent interaction. The specific amino-acid residues and the relative contributions of direct binding and multiprotein-complex formation remain undefined.

### 2.4 Regulation of CFH expression and CFH-derived circular RNAs

CFH abundance in cancer cells is influenced by signaling and epigenetic mechanisms. In cutaneous squamous cell carcinoma, CFH and FHL-1 were upregulated, and their silencing reduced proliferation, migration, and basal ERK1/2 activity (33). Inhibition of ERK1/2 or p38 also reduced CFH and FHL-1 expression, indicating reciprocal regulation between MAPK signaling and these proteins.

In lung cancer cells, STAT4 expression was associated with CFH expression and secretion. STAT4 bound directly to the CFH promoter, whereas restoration of SOCS1 or SOCS3 reduced CFH promoter activity and protein expression (34). Reduced SOCS1/3 expression was not attributed to methylation of their own promoters; instead, differential methylation was identified within regulatory regions of STAT4 (34).

In HCC, integrated methylation and transcriptomic analyses linked increased methylation at CFH CpG sites and reduced CFH mRNA expression with postoperative recurrence (35). In GBM, bioinformatic analysis identified CFH among methylation-associated differentially expressed genes and incorporated CFH, CASP1, and TTLL7 into a prognostic signature (36).

The CFH variant NM_000186.4:c.2314G>A, corresponding to p.Asp772Asn (D772N), was identified in a multicancer pedigree together with multiple additional genomic and epigenomic alterations (37). Its occurrence within this broader molecular background limits its interpretation as an independent determinant of cancer susceptibility.

Circular transcripts from the CFH locus have also been implicated in cancer. circ-CFH regulated the miR-377-3p/RNF38 axis in HCC and the miR-149/AKT1 axis in glioma (38, 39). These non-coding RNA mechanisms are distinct from the functions of the FH protein encoded by linear CFH mRNA.

### 2.5 Local and cell-autonomous FH functions outside cancer

Studies outside cancer provide additional examples of locally produced and cell-autonomous FH functions. In kidney endothelial cells, FH deficiency disrupted cytoskeletal organization and monolayer integrity, increased proliferation, impaired mitochondrial respiration, and enhanced inflammatory and complement-related signaling; several changes were reduced by FH reconstitution (40). In retinal pigment epithelial cells, CFH loss was accompanied by NF-κB activation and altered inflammatory and complement-related gene expression, whereas extracellular FH, C3, or C3b did not reproduce or reverse the NF-κB phenotype (41).

Myeloid CFH deletion increased intracellular C3 consumption and macrophage efferocytosis without corresponding C5 activation in experimental atherosclerosis (42). In glomerular endothelial cells exposed to inflammatory stimulation, reduced intracellular FH was associated with increased extracellular FH secretion, enhanced SAA1–TLR2–NF-κB signaling, and impaired barrier function (43). Mesangial-cell-derived FH regulated complement deposition, proliferation, Cdc42 expression, actin organization, and cell motility, while exogenous FH did not reproduce the effects of endogenous FH (44). Global FH deficiency was also associated with altered bone architecture and osteoblast–osteoclast activity (45). The relevance of these tissue-specific mechanisms to cancer requires validation in individual tumor models.

## 3 FH at the tumor–host immune interface

**Figure 3:**
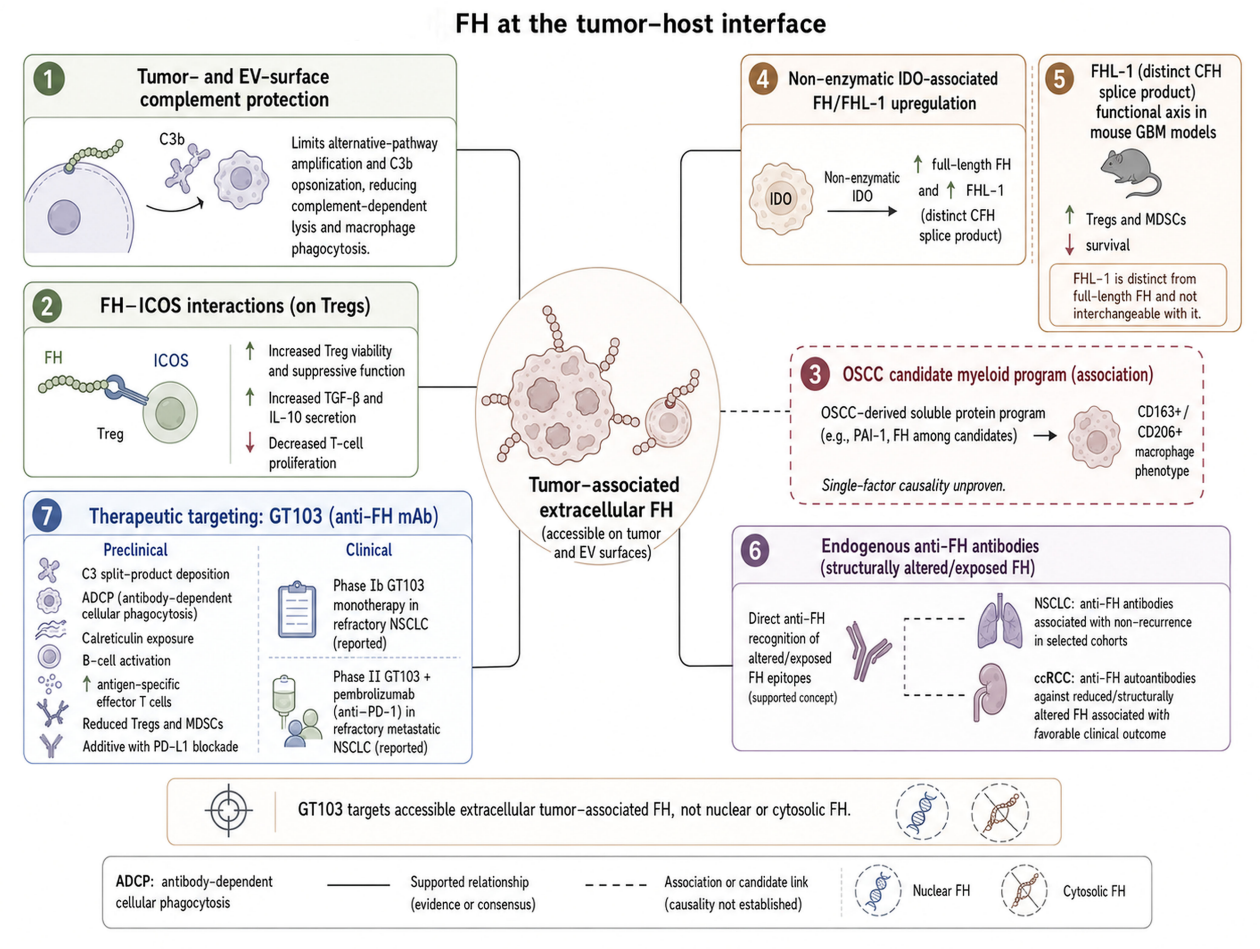
FH at the tumor–host interface. Tumor-associated extracellular FH accessible on tumor and EV surfaces limits alternative-pathway amplification and C3b opsonization, reducing complement-dependent lysis and macrophage phagocytosis. FH binding to ICOS supports Treg viability and suppressive function, increases TGF-β and IL-10 secretion, and reduces T-cell proliferation. Non-enzymatic tumor-cell IDO increases full-length FH and FHL-1 in glioblastoma; FHL-1, a distinct CFH splice product, increased Tregs and MDSCs and reduced survival in mouse GBM models. In OSCC, FH was identified among multiple candidate soluble mediators of a CD163+/CD206+ macrophage phenotype, but single-factor causality remains unproven. Endogenous anti-FH antibodies recognize structurally altered or selectively exposed FH epitopes and show context-dependent clinical associations in NSCLC and ccRCC. GT103 recognizes a conformationally distinct, accessible extracellular tumor-associated FH epitope and induces C3 split-product deposition, antibody-dependent cellular phagocytosis (ADCP), calreticulin exposure, B-cell activation, increased antigen-specific effector T cells, reduced Tregs/MDSCs, and additive activity with PD-L1 blockade in preclinical models. Phase Ib GT103 monotherapy and phase II GT103 plus pembrolizumab have been reported in refractory NSCLC. GT103 does not directly target nuclear or cytosolic FH. Solid lines indicate supported relationships; dashed lines indicate associations or candidate links for which causality is not established. ccRCC, clear-cell renal cell carcinoma; EV, extracellular vesicle; GBM, glioblastoma; IDO, indoleamine 2,3-dioxygenase; MDSC, myeloid-derived suppressor cell; NSCLC, non-small cell lung cancer; OSCC, oral squamous cell carcinoma; Treg, regulatory T cell.

Within tumors, FH may be produced by malignant or stromal cells or recruited from the circulation. Similar protein-detection patterns may therefore reflect local synthesis or deposition of circulating protein, making it important to distinguish tissue CFH transcription from cell-surface- or extracellular-vesicle-associated FH. Circulating anti-FH antibodies provide a related but biologically distinct measure of host humoral recognition. The major extracellular, immune-cell, humoral, and therapeutic relationships at this interface are summarized in **Figure 3**.

### 3.1 Local extracellular complement regulation

A well-characterized extracellular function of FH in cancer is the protection of tumor-associated surfaces from alternative-pathway amplification (21, 46). FH was detected on extracellular vesicles derived from several tumor cell lines and on circulating extracellular vesicles from patients with metastatic NSCLC (21). On tumor-derived exosomes, FH reduced complement-dependent lysis and macrophage phagocytosis, and higher levels of FH-containing vesicles were associated with greater metastatic potential among the tested tumor cell lines (21).

### 3.2 Myeloid-cell and regulatory T-cell interactions

Conditioned media from the H413 and TR146 oral squamous cell carcinoma cell lines promoted the differentiation of human monocytes into macrophages characterized by increased CD163 and CD206 and reduced CD11c, CD86, and HLA-DR expression (47). CD25 expression was also increased in macrophages conditioned with H413-derived medium. These H413-conditioned macrophages failed to activate allogeneic T cells and inhibited CD3/CD28-induced T-cell activation and proliferation (47). Fractionation experiments attributed macrophage differentiation primarily to soluble proteins rather than small metabolites or extracellular vesicles, and differential proteomic analysis identified FH, plasminogen activator inhibitor 1, and other proteins as potential mediators of the CD163^+^CD206^+^ phenotype (47).

In glioblastoma, a non-enzymatic function of tumor-cell IDO increased CFH and FHL-1 expression independently of tryptophan catabolism, and higher intratumoral levels of both proteins were associated with shorter survival (24). In mouse GBM models, expression of FHL-1 by IDO-deficient tumor cells increased intratumoral Tregs and MDSCs and reduced survival (24).

A separate study identified FH as an ICOS ligand in human and murine glioma models (25). FH binding to ICOS increased Treg viability and suppressive function, enhanced TGF-β and IL-10 secretion, and inhibited T-cell proliferation (25). Glioma cells produced FH, and increased CFH expression was associated with greater Treg abundance and poorer clinical outcome (25).

### 3.3 B-cell biology and endogenous anti-FH antibodies

In non-tumor Cfh-deficient mice, uncontrolled splenic C3 consumption was accompanied by accumulation of marginal-zone B cells, increased BTK and SYK phosphorylation, and elevated CR2 expression on mature splenic B cells (48). Aged Cfh-deficient mice also developed splenomegaly, germinal-center hyperactivity, and increased anti-double-stranded-DNA antibodies (48).

In NSCLC, anti-FH autoantibodies were detected more frequently in early-stage than in advanced disease (27). In a separate stage I cohort, antibody positivity was associated with a lower cumulative incidence of recurrence over 60 months, whereas changes in antibody levels during the first postoperative year were not associated with recurrence (26).

The anti-FH autoantibody and GT103 recognize the PIDNGDIT epitope within SCR19 of FH (49). Patient plasma antibodies showed weak cross-reactivity with a related human metapneumovirus peptide, whereas GT103 recognized peptides from human metapneumovirus, HERV-K, and measles virus with lower affinity than the corresponding FH epitope (49). These findings are consistent with molecular mimicry as a possible contribution to the anti-FH response (49).

In ccRCC, autoantibodies against a reduced form of FH, corresponding to a structurally altered tumor-associated antigen, were associated with favorable clinical outcome (50).

### 3.4 Therapeutic targeting of tumor-associated FH with GT103

GT103 is a fully human IgG3 monoclonal antibody cloned from peripheral B cells of patients carrying the anti-FH autoantibody (46, 51). It recognizes a conformationally distinct epitope within SCR19 that is displayed by tumor-associated FH but is not exposed on native soluble FH or normal tissues (46, 51).

GT103 triggered classical-pathway lysis and antibody-dependent macrophage phagocytosis of tumor-derived exosomes (21). In tumor-cell models, it induced C3 split-product deposition, antibody-dependent cellular phagocytosis, calreticulin translocation to the plasma membrane, and B-cell activation; its antitumor activity *in vivo* was dependent on B cells (46).

In syngeneic tumor models, murinized GT103 reduced intratumoral Tregs and MDSCs, increased antigen-specific effector T cells, and produced an additive antitumor effect with PD-L1 blockade (51).

In a phase Ib dose-escalation study, 31 patients with refractory NSCLC received GT103 (31). The maximum tolerated dose was not reached, no objective responses were observed, and nine patients achieved stable disease. GT103 at 10 mg/kg every three weeks was selected as the recommended phase II dose (31).

The subsequent single-arm phase II study used a Simon two-stage design to evaluate GT103 at 10 mg/kg combined with pembrolizumab at 200 mg every three weeks in patients with refractory metastatic NSCLC (32). The study enrolled 21 patients and observed two objective responses—one complete response and one partial response—corresponding to an objective response rate of 10% (32). The disease-control rate was 67%, and four patients maintained disease control for more than nine months. Median progression-free survival was 2.6 months and median overall survival was 10.3 months. The combination was generally well tolerated, with no new safety signals (32).

## 4 Expression patterns, systemic context, and biomarkers

**Figure 4:**
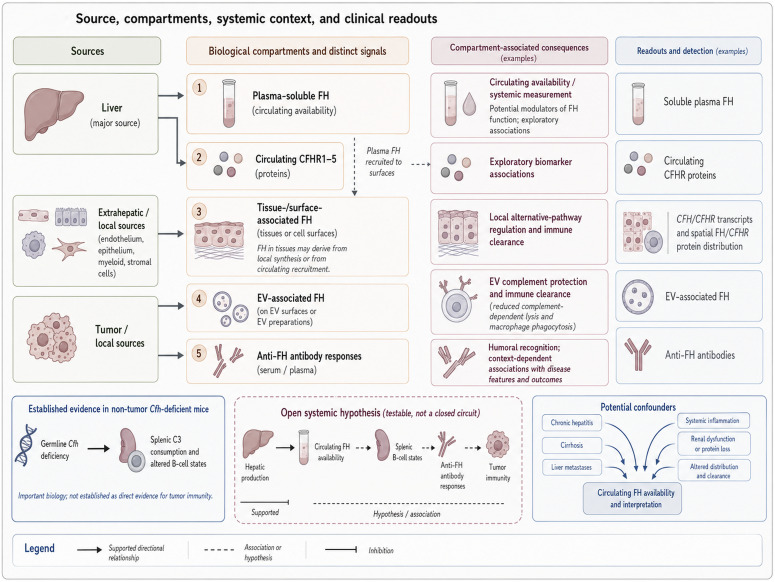
Source, compartments, systemic context, and clinical readouts of FH biology. The liver is the major source of plasma-soluble FH and circulating CFHR proteins. Extrahepatic endothelial, epithelial, myeloid, stromal, and tumor cells contribute local FH pools, and tumor/local sources contribute EV-associated FH. Tissue- or surface-associated FH may arise from local synthesis or recruitment from plasma. Plasma-soluble FH represents circulating availability and a systemic measurement; tissue- or surface-associated FH supports local alternative-pathway regulation and immune clearance; EV-associated FH reduces complement-dependent lysis and macrophage phagocytosis; CFHR proteins and anti-FH antibody responses provide distinct exploratory biomarker and humoral signals. Non-tumor germline Cfh deficiency and its splenic C3/B-cell phenotype are shown as a separate established branch, not as direct evidence for tumor immunity. The proposed systemic sequence from hepatic production to circulating FH availability, splenic B-cell states, anti-FH responses, and tumor immunity remains an open, testable hypothesis; only hepatic production to circulating FH is shown as supported. Chronic hepatitis, cirrhosis, liver metastases, systemic inflammation, renal dysfunction/protein loss, and altered distribution/clearance may confound circulating FH interpretation. Compartment-specific readouts include soluble plasma FH, circulating CFHR proteins, CFH/CFHR transcripts, spatial FH/CFHR protein distribution, EV-associated FH, local airway fluids, urine, and anti-FH antibody assays. Solid arrows indicate supported directional relationships; dashed arrows indicate associations or hypotheses. BALF, bronchoalveolar lavage fluid; CFHR, complement factor H-related protein; EV, extracellular vesicle.

CFH and CFHR expression patterns vary across tissues, tumor lineages, cellular sources, and disease states. Because complement can either restrain or support tumor progression according to its spatial, temporal, and therapeutic context, an increase or decrease in a single FH-family member does not carry a uniform biological meaning (8). **Figure 4** organizes these findings according to source, biological compartment, systemic modifiers, and compartment-specific readouts.

### 4.1 Source and tissue context

The liver is a major source of circulating complement proteins, including members of the FH protein family (14, 52, 53). FH is also produced by extrahepatic endothelial, epithelial, myeloid, and tumor cells, and local production has been reported for selected FHR proteins (14). Tissue transcript abundance, tissue-associated protein, and circulating protein concentration therefore represent distinct measurements of FH-family biology. Tumor- and compartment-specific expression patterns, functional findings, and evidence boundaries are summarized in **Table 2**.

**Table 2:** Tumor- and compartment-specific patterns of FH-family biology in cancer.

Tumor setting	FH-family entity/compartment	Principal finding	Evidence boundary	Ref(s)
cSCC	FH and FHL-1; tumor-cell/tissue expression	Both were upregulated; silencing reduced proliferation, migration, and basal ERK1/2 activity, while ERK1/2 or p38 inhibition reduced their expression.	Cellular and tissue evidence; serum associations are a separate clinical signal.	(33)
ccRCC and lung cancer models	Intracellular FH; lysosome-positive localization reported	CFH silencing produced cell-type-dependent effects on proliferation, migration, cell death, p53 phosphorylation, and NF-κB nuclear translocation.	Localization does not establish a lysosomal site of action; several tested cell types were nonresponsive.	(19)
ccRCC cells and fibroblasts	FH; nuclear and cytosolic intracellular pools	Nuclear FH–E2F3 interactions were linked to p53 and cell-cycle regulation; cytosolic FH was associated with CapZ-dependent actin organization.	Distinct compartments; exact interface residues remain unresolved and CapZ binding was assay dependent.	(20)
Lung cancer	CFH; tumor-cell expression and secretion	STAT4 bound the CFH promoter, whereas restoration of SOCS1 or SOCS3 reduced promoter activity and FH expression.	The pathway has effects beyond CFH and does not by itself establish clinical dependence on FH.	(34)
GBM/glioma	FH and FHL-1; locally produced extracellular proteins	Non-enzymatic IDO increased CFH/FHL-1; FHL-1 promoted Treg/MDSC accumulation in mouse models, and FH directly engaged ICOS on Tregs.	FH and FHL-1 findings arose from distinct experiments and are not interchangeable mechanisms.	(24, 25)
OSCC	FH within a soluble protein secretome; conditioned medium	Tumor-conditioned media induced CD25+CD163+CD206+ macrophages with reduced T-cell stimulatory capacity; FH was identified among candidate soluble mediators.	A multi-protein secretome was tested; single-factor causality for FH was not established.	(47)
Tumor EVs and metastatic NSCLC	FH; EV surface	EV-associated FH reduced complement-dependent lysis and macrophage phagocytosis.	Vesicular FH must be distinguished analytically from abundant residual soluble plasma FH.	(21)
HCC	CFH, CFHR3, CFHR1 (reported as CFHL1), and CFHR4; tumor tissue	Reduced expression, methylation, hypoxia-related signatures, and outcome associations were reported across studies.	Tissue findings are not surrogates for circulating FH or splenic immune effects; evidence is mainly retrospective/transcriptomic.	(35, 54–59)
Cholangiocarcinoma	CFHR3; tumor tissue expression	Low CFHR3 expression was associated with survival and immune-infiltration patterns.	Retrospective/transcriptomic evidence; no direct circulating-protein inference.	(60)
HGSOC	FH; serum EV-associated protein	EV-CFH differed between platinum-resistant and platinum-sensitive disease and contributed to a high-performing combined EV panel.	Biomarker association does not establish causal platinum resistance; EV methods affect interpretation.	(28)

cSCC, cutaneous squamous cell carcinoma; ccRCC, clear-cell renal cell carcinoma; EV, extracellular vesicle; FHL-1, factor H-like protein 1; GBM, glioblastoma; HCC, hepatocellular carcinoma; HGSOC, high-grade serous ovarian cancer; NSCLC, non-small cell lung cancer; OSCC, oral squamous cell carcinoma; Treg, regulatory T cell. Reference numbers correspond to the main manuscript.

In HCC, integrated methylation and transcriptional profiling linked increased CFH CpG methylation and reduced CFH mRNA expression with postoperative recurrence (35). CFHR3 downregulation was associated with miR-590-3p-dependent regulation of STAT3 and p53 signaling (54), hypoxia-related tumor phenotypes (55), and poorer clinical outcomes in several transcriptomic and prognostic analyses (55–57). Reduced expression of CFHR1, termed CFHL1 in the original report, and CFHR4 was also associated with unfavorable outcomes in HCC (58, 59). Low CFHR3 expression has likewise been reported in cholangiocarcinoma and associated with survival and immune-infiltration patterns (60).

These hepatobiliary studies primarily examined tumor-tissue transcripts or proteins. Because circulating FH-family proteins also reflect hepatic synthesis, tissue recruitment, distribution, and clearance, changes within tumor tissue may not be accompanied by proportional changes in plasma.

### 4.2 Circulating, extracellular-vesicle-associated, and humoral signals

FH itself can be measured in soluble plasma and extracellular-vesicle-associated compartments, whereas anti-FH antibodies constitute a separate humoral signal reflecting host recognition of FH epitopes. Tumor-derived extracellular vesicles can acquire FH on their surfaces (21). In high-grade serous ovarian cancer, EV-associated CFH and TMEM205 differed between platinum-resistant and platinum-sensitive disease. EV-CFH yielded an area under the receiver operating characteristic curve of 0.95, whereas the combined CFH–TMEM205 model yielded an area under the curve of 0.973 (28).

Soluble FH, EV-associated FH, and anti-FH antibodies represent different biological signals. EV-associated measurements may reflect vesicle surface composition, whereas anti-FH antibodies reflect host recognition of FH epitopes rather than the amount of circulating FH (26, 27, 50). Because soluble FH is abundant in plasma, EV-associated findings also depend on isolation and analytical procedures capable of distinguishing vesicular FH from residual soluble protein.

Circulating measurements may additionally be influenced by hepatic synthesis, renal handling or protein loss, systemic inflammation, tissue recruitment, and clearance (8, 14–18, 52, 53). Clinical studies should therefore record chronic hepatitis, cirrhosis, liver metastases, systemic inflammation, renal dysfunction, and other determinants of protein loss or clearance when interpreting circulating FH. These factors complicate direct comparisons between tumor-tissue expression and circulating FH-family proteins, particularly in hepatobiliary cancers or in patients with hepatic or renal dysfunction.

### 4.3 Biomarker evidence across biological compartments

The principal clinical biomarker studies across biological compartments are summarized in **Table 3**. In the study by Daugan et al. (19), intracellular FH staining, but not membranous FH staining, was associated with poor prognosis in clear-cell renal cell carcinoma and lung adenocarcinoma, whereas no comparable prognostic association was observed in lung squamous cell carcinoma. This localization-dependent pattern supports interpreting tissue FH staining by subcellular compartment rather than as a single aggregate biomarker. Local-fluid studies have reported increased CFH in bronchoalveolar lavage fluid and sputum from patients with lung cancer (22). In bladder cancer, analytical platforms have been developed for urinary CFHR1 detection using electrochemical ELISA and electrochemiluminescence-based immunosensors (61, 62). The bladder tumor antigen assay illustrates an important preanalytical limitation: hematuria can introduce blood-derived FH-related antigens into urine and produce positive findings in both malignant and non-malignant conditions (63).

**Table 3:** Clinical biomarker evidence for FH-family analytes across biological compartments.

Clinical setting	Analyte/sample	Clinical association and quantitative result	Main limitation	Ref(s)
Early-stage NSCLC	Serum anti-FH autoantibodies	More frequent in early than advanced disease; positivity in a stage I cohort was associated with lower 60-month recurrence, whereas first-year titer changes were not associated with recurrence.	Separate cohorts and assays; no universal cutoff and the biological source of the response remains undefined.	(26, 27)
ccRCC	Serum antibodies to reduced/structurally altered FH	Associated with favorable clinical outcome in exploratory complementomic analysis.	Disease- and assay-specific; external validation is required.	(50)
HGSOC	Serum EV-CFH ± TMEM205	Discriminated platinum-resistant from platinum-sensitive disease; AUC 0.950 for EV-CFH and 0.973 for the combined model.	Small retrospective cohorts and EV-isolation dependence; no causal inference.	(28)
Advanced cSCC	Pretreatment serum FH	Higher in advanced than high-risk disease; lower baseline FH was associated with longer PFS on cemiplimab, while on-treatment FH was not associated with response. Adding clinical variables improved classification.	Retrospective single-center study without external validation; not a stand-alone predictive biomarker.	(66)
SCLC	Serum FH, C3, C4, and clinical variables	Lower FH was associated with greater cancer-related mortality; the combined model achieved an internally validated C-statistic of 0.905.	Retrospective cohort with internal, not external, validation.	(30)
Lung cancer	BAL fluid and sputum FH	FH was elevated and proposed as an adjunct local-fluid marker.	Not established as a stand-alone diagnostic test; local-fluid preanalytics require control.	(22)
Bladder cancer	Urinary CFHR1 and BTA-related FH antigen	Electrochemical platforms detected CFHR1; hematuria can introduce blood-derived FH-related antigen and produce positive BTA results in malignant and non-malignant conditions.	Sensor-development studies and major hematuria confounding.	(61–63)
Ovarian cancer	Sialylated serum FH	Altered FH glycosylation was reported in a discovery-phase serum study.	Assay-specific and not yet a validated clinical discriminator.	(64)
Ovarian cancer	Tissue CFH expression	Identified among candidate diagnostic expression markers by bioinformatics with tissue qPCR validation.	Tissue expression study rather than liquid-biopsy validation.	(65)
Prostate cancer	CAF-associated FH	Identified as a stromal tissue marker through comparative fibroblast profiling.	Not a circulating biomarker; disease-specific validation is required.	(23)
HCC	Tumor-tissue CFH/CFHR expression and signatures	Associated with recurrence or survival across methylation, transcriptomic, and prognostic studies; no common threshold was established.	Retrospective/bioinformatic evidence; chronic liver disease and tissue–plasma discordance complicate interpretation.	(35, 54–59)
Cholangiocarcinoma	Tumor-tissue CFHR3	Low expression was associated with poor outcome and immune-infiltration patterns.	Retrospective transcriptomic evidence; no validated circulating threshold.	(60)
Pituitary adenoma	CFH variants and serum FH	rs1061170 was associated with hormonal activity and invasiveness in an exploratory study; serum FH was not a validated discriminator.	Small disease-specific genetic association study.	(29)

AUC, area under the receiver operating characteristic curve; BAL, bronchoalveolar lavage; BTA, bladder tumor antigen; CAF, cancer-associated fibroblast; cSCC, cutaneous squamous cell carcinoma; ccRCC, clear-cell renal cell carcinoma; EV, extracellular vesicle; HCC, hepatocellular carcinoma; HGSOC, high-grade serous ovarian cancer; NSCLC, non-small cell lung cancer; PFS, progression-free survival; SCLC, small cell lung cancer. Reference numbers correspond to the main manuscript.

Circulating and tissue-based studies have examined different CFH-related analytes. Altered sialylation of serum CFH was reported in ovarian cancer (64), whereas an independent bioinformatic study with tissue qPCR validation identified CFH among several potential diagnostic expression markers for ovarian cancer (65). In prostate cancer, CFH identified through comparative profiling of cancer-associated fibroblasts represents a stromal tissue marker rather than a circulating analyte (23). A smaller pituitary adenoma study examined CFH rs1061170/rs1410996 and serum FH, reporting genotype associations with hormonal activity and invasiveness but not establishing serum FH as a validated discriminator (29).

In a retrospective cutaneous squamous cell carcinoma cohort, pretreatment serum CFH was higher in advanced than in high-risk disease. Among patients with advanced disease receiving cemiplimab, lower baseline CFH was associated with longer progression-free survival, whereas CFH measurements during treatment were not associated with response. Serum CFH alone provided limited separation between high-risk and advanced disease, while a model incorporating CFH and clinical variables showed better classification performance (66).

In SCLC, lower serum CFH was associated with a higher risk of cancer-related death. A prognostic model incorporating CFH, C3, C4, and clinical variables achieved an internally validated C-statistic of 0.905 (30).

The EV-CFH–TMEM205 panel in ovarian cancer (28), the serum CFH–clinical variable model in cutaneous squamous cell carcinoma (66), and the CFH–C3–C4 model in SCLC (30) illustrate how the potential value of FH-related biomarkers depends on the disease setting, biological compartment, and accompanying variables. Current evidence therefore supports context-specific biomarker combinations rather than a universal CFH threshold applicable across tumor types.

## 5 Clinical implications and research priorities

Clinical translation of FH biology requires alignment between the biological compartment in which FH acts and the intervention used to modify that function.

### 5.1 Compartment-specific therapeutic implications

GT103 recognizes accessible tumor-associated FH on extracellular surfaces, whereas the FH–E2F3 and FH–CapZ interactions involve nuclear and cytosolic FH, respectively (20, 21, 46, 51). Antibody-based targeting of tumor-surface FH would therefore not be expected to directly modify these intracellular functions.

MAPK and STAT4 signaling can regulate CFH expression in cutaneous squamous cell carcinoma and lung cancer, respectively (33, 34). Interventions directed at these upstream pathways may consequently alter FH abundance, but their effects would extend beyond CFH itself. Extracellular tumor-associated FH can be addressed with antibodies such as GT103, whereas intracellular FH mechanisms would require strategies capable of entering the relevant cellular and subcellular compartments.

### 5.2 Remaining mechanistic questions

The available structural and biochemical evidence has begun to define the intracellular interactions of FH. CCP6–8 was implicated in E2F3 binding under physiological ionic-strength conditions, whereas CAPZB-associated binding involved CCP6–8 and the C-terminal CCP18–20/CCP19–20 regions under assay-dependent conditions (20). Intracellular FH also showed a lower apparent molecular mass than plasma-derived FH, consistent with reduced glycosylation (20).

The remaining questions concern the precise residues forming these interfaces, whether the observed interactions occur directly at endogenous protein abundance, and how intracellular FH reaches or is retained within cytosolic and nuclear compartments. Targeted mutagenesis, endogenous protein tagging, and compartment-specific rescue experiments would allow these points to be examined without assuming that all proteins detected within the same complex interact directly.

### 5.3 Experimental and clinical validation

Conditional and tissue-specific CFH models are needed to distinguish local tumor-associated functions from the systemic changes observed in germline Cfh deficiency and from cell-autonomous phenotypes identified in non-tumor tissues (40–45, 48). In clinical studies, paired tissue and blood sampling could integrate measurements of tumor CFH/CFHR expression and spatial localization with assessments of soluble and EV-associated FH, anti-FH antibodies, and hepatic and renal function.

A graded liver-specific CFH model, followed by time-resolved assessment of circulating FH, splenic B-cell states, anti-FH antibodies, and tumor immune phenotypes, would provide a direct way to test whether these compartments change independently or in coordination. Such a design would directly test whether changes in these compartments occur independently or in coordination without presupposing a continuous causal pathway.

GT103 provides a clinical example of targeting a defined extracellular FH compartment. Phase Ib and phase II studies have established the feasibility of administering GT103 in patients with refractory NSCLC, while determinants of target engagement and clinical activity remain incompletely defined (31, 32). Future studies should therefore evaluate tumor-surface FH epitope abundance, complement competence, Fc-effector context, baseline anti-FH antibodies, and tumor lineage alongside treatment outcomes.

## 6 Conclusions

FH contributes to cancer through intracellular mechanisms, local tumor–host interactions, and source-dependent tissue and circulating protein pools. Intracellularly, nuclear FH–E2F3 interactions and cytosolic FH–CapZ-associated regulation link FH to cell-cycle control and actin organization, whereas localization to lysosome-positive compartments alone does not identify a lysosomal site of action. At the tumor–host interface, extracellular FH protects tumor-cell and extracellular-vesicle surfaces from complement-mediated injury and participates in selected myeloid-cell, regulatory T-cell, and humoral immune responses. Structural alteration or selective exposure of tumor-associated FH can also permit recognition by endogenous anti-FH antibodies and by the therapeutic antibody GT103. Across tissues and the circulation, hepatic and extrahepatic production, local recruitment, extracellular-vesicle association, distribution, and clearance shape biologically distinct FH-family pools.

Together, these observations support a source- and compartment-resolved hypothesis of FH biology in cancer. Systemic production contributes to the FH available to tissues, whereas tumor and stromal cells further determine its local synthesis, surface recruitment, intracellular localization, and structural state. These processes may allow FH to participate in extracellular complement regulation, intracellular signaling, immune-cell interactions, and antibody recognition in different biological compartments. The hypothesis is derived from studies conducted in different tumor types, non-tumor models, and experimental settings, and the proposed links among circulating FH, splenic B-cell states, anti-FH responses, and tumor immunity have not been demonstrated as a continuous causal sequence. Paired longitudinal measurements and graded tissue-specific manipulation of CFH will be required to define the direction, strength, and tumor specificity of these relationships.
